# Effects of one single-dose methylphenidate compared to one single-dose placebo on QbTest performance in adults with untreated ADHD: a randomized controlled trial

**DOI:** 10.1186/s12888-023-05231-8

**Published:** 2023-10-17

**Authors:** Lennart Jansson, Monica Löhman, Mona Östlund, Blanca Domingo

**Affiliations:** 1 Psychiatric Clinic, Region Västmanland, 721 89 Västerås, Sweden; 2 Centre for Clinical Research, Uppsala University, Västerås, Sweden

**Keywords:** ADHD, Adult, Methylphenidate, Placebo, Randomized trial, Continuous performance tests (CPT)

## Abstract

**Background:**

Treatment of adults with attention-deficit/hyperactivity disorder (ADHD) primary involves methylphenidate (MPH). Earlier studies have identified placebo responders to increase toward the end of the treatment periods. However, little is known about the immediate effects of placebo on the core symptoms of ADHD in adults. The present study aimed to examine the effects of one single-dose MPH compared to one single-dose placebo during clinical assessments with continuous performance tests (CPT).

**Methods:**

In a randomized study with cross-over design, 40 adults between 19 and 64 years (72.5% women) with untreated ADHD were consecutively enrolled. The study comprised two trial days with four days in between. The QbTest was performed twice on the same day, before and 80 min after intake of one single-dose 20 mg immediate release methylphenidate (IR-MPH) and with one single-dose placebo, in randomized order.

**Results:**

Performance improved in QbInattention, F (3, 117) = 38.25, *p* < 0.001, after given IR-MPH (mean diff = 1.14) and after placebo (mean diff = 0.60) with the effect sizes 1.17 and 0.63 respectively. IR-MPH improved performance in QbActivity (mean diff = 0.81, *p* < 0.001) and QbImpulsivity (mean diff = 0.46, *p* < 0.04). The proportion of improvements (a decrease by ≥ 0.5 Qb-score) in the parameters QbInattention, QbActivity and QbImpulsivity were 90%, 60% and 52.5%, respectively. After given placebo, corresponding proportions were 60%, 30% and 35%, respectively.

**Conclusions:**

There seems to be an immediate placebo response in the core symptom inattention. The effect of placebo cannot be ruled out and must be taken in consideration during drug trials with continuous performance tests (CPTs).

**Trial registration:**

ClinicalTrials.gov; Identifier: NCT02473185.

## Introduction

Attention-deficit/hyperactivity disorder (ADHD) is a neurodevelopmental syndrome with onset during childhood and often persists into adulthood [1–3]. The core ADHD symptoms include a frequent and persistent pattern of inattention and/or hyperactivity-impulsivity that interferes with functioning in daily living [4]. In adults, some studies have reported that inattention and executive dysfunction becomes more prominent while impulsivity remains problematic and hyperactivity decreases [5, 6]. The prevalence of ADHD in adults is in the range 2%-5% worldwide [7–9]. In Sweden, the number of clinical adult patients diagnosed with ADHD increased from 0.58 per 1,000 persons in 2006 to 3.54 per 1,000 persons in 2011 [10]. In a Swedish study of outpatients in general psychiatric care, 22% were diagnosed with ADHD in adulthood [11]. Parallelly, the number of patients in need of support and treatment increases.

The treatment of adults with ADHD should follow a multimodal and multidisciplinary approach (e.g., psychoeducation, cognitive behavior therapy, coaching for ADHD and pharmacotherapy [5]). However, many adults request pharmacological treatment. One of the most common pharmacological treatments of ADHD in adults involves primary methylphenidate (MPH) [12]. MPH is a psychostimulant that blocks the reuptake of norepinephrine and dopamine and improves the symptoms and impairing behaviours associated with ADHD. MPH is provided in different formulations e.g., immediate release (IR-MPH) and extended release (ER-MPH). Numerous randomized, double blind, placebo-controlled treatment studies have explored positive effects of MPH in adults with ADHD [13–17]. In most of the studies, the patients were given osmotic-release oral system methylphenidate (OROS-MPH) or (ER-MPH) in doses up to 1.3 mg/kg/day [13, 14]. However, placebo responders have been reported in the range of 39%-46%, depending on the primary outcome measures chosen and differences in duration of follow-up [13, 15, 18].

The placebo effect is well known and a clinically important phenomenon in the patient’s treatment. Extensive research has been conducted to elucidate this [19, 20].

Most placebo-controlled studies have reported subjective outcomes (i.e., clinical assessments and self-report scales). Although Biederman et al., [21] have found strong correlations between clinician-assessed ADHD symptoms and patients self-reports, many patients have difficulties judging if their medical treatment has any effect [21, 22]. Self-assessment instruments are often too non-specific, and thereby too inclusive, because many patients without ADHD may rate themselves highly on these scales [23, 24].

One approach to improve assessment in ADHD is to supplement clinical judgement with computerized continuous performance tests (CPTs). The CPT is a neuropsychological assessment tool that provides an objective and standardized method for assessing attention and impulsivity. It eliminates subjective biases that can occur in self-report measures and provides quantifiable data. The CPT may be useful for monitoring the effects of ADHD treatment interventions.

It is often a challenge to meet the patients’ requests for drug treatment. The medical staff needs support in their assessments to evaluate the effect of the drug for each patient. In contrast to patients’ subjective self-reports, it would be useful to have an objective tool to assess the patients’ level of response in different core signs. MPH is available as immediate release (IR-MPH), which could be suitable for medical evaluation. By offering the patient IR-MPH together with a CPT, a relatively quick response is made possible. The assessment and the results from the objective measurement can make it easier to offer adequate long-term treatment for each patient. One of several CPTs is the QbTest which is developed to measure the core symptoms of ADHD and can be used when to start a pharmacological treatment with a new patient [25]. Bijlenga et al., concluded that the QbTest is more sensitive to medication effects than the ADHD Rating Scale (ADHD-RS) [26].

Placebo responses in earlier studies were found to increase toward the end of the treatment periods [27, 28]. However, little is known about the immediate effects of placebo on the core symptoms of ADHD in adults. Do they already occur during the first drug trial with CPTs? Increased knowledge of the impact of the placebo response on the core symptoms may improve decisions about which treatment is most advantageous. It is therefore relevant to analyse the effects of placebo on performance in the ADHD core symptoms, hyperactivity, inattention and impulsivity.

The aim of the present study was to examine the effects of one single-dose IR-MPH compared to one single-dose placebo on performance in ADHD core symptoms during clinical assessments in adults with untreated ADHD. We assumed that IR-MPH would improve participants´ performance in the cardinal parameters QbActivity and QbInattention. Regarding placebo, we hypothesized that the placebo response would be lower compared to IR-MPH but effective in all three core symptoms. The placebo response would be higher at the beginning of the task, then decrease towards the end because adults with ADHD often have difficulty focusing for a longer period. Throughout this paper, we will use the term “placebo response” as to the outcome of a clinical trial.

## Material and methods

### Sample

The participants were remitted from six general psychiatric outpatient units between October 2015 and May 2018, to a neuropsychiatric investigation at the Psychiatric Clinic in the County of Västmanland, Sweden. In this period 105 new patients visit the clinic. Each individual was involved in a neuropsychological and neuropsychiatric assessment made by a team of clinical professionals having a solid professional experience in the field of neurodevelopmental disorders. Inclusion criteria for this study were: (a) 18 years old or older, (b) ADHD were diagnosed according to the DSM-5 criteria [4], (i.e. the presence of at least five symptoms for inattention and/or at least five symptoms for hyperactivity/impulsivity), based on clinical interviews (the Diagnostic Interview for ADHD in Adults Version 2—DIVA 2.0 [29], the Mini International Neuropsychiatric Interview – MINI [30, 31], the self-report questionnaires (the Adult Self Report Scale – ASRS ver 1.1 [32], and the Wender Riktad ADHD-Symtom Skala – WRASS). The WRASS is a Swedish adaptation of the Wender-Reimerr Adult Attention-Deficit Disorder Scale (WRASDDS) [33]. History of childhood symptoms were assessed with the Wender Utah Rating Scale – WURS [34, 35] and information from the participant´s parents or other close relatives was collected by telephone interview, (c) Q-score ≥ 1.3 on at least one of the cardinal parameters QbActivity, QbInattention or QbImpulsivity on the QbTest. Main exclusion criteria were: tested positive for alcohol or drugs during the last month, untreated comorbid psychiatric or somatic illness, blood pressure ≥ 150/90 mm Hg, irregular pulse or pulse ≥ 100 bpm, and tested positive for pregnancy. Those adults who met the inclusion criteria and not the exclusion criteria were consecutively invited to participate in the study.

### Measures

#### The Quantified Behavioral Test

The Quantified Behavioral Test (QbTest; QbTech Ltd, www.qbtech.com) is a computerized CPT including measures of inattention and impulsivity combined with a motion tracking device recording activity measure. The QbTest measures the three cardinal symptoms of ADHD; hyperactivity, inattention, and impulsivity presented in the test report as cardinal parameters – QbActivity, QbInattention, and QbImpulsivity. The Qb-scores are normalized standard scores, which are adjusted for age and gender effects. In the general population, the Qb-scores have a mean = 0, and an SD = 1. Higher scores indicate more severe symptoms. A Qb-score within the range from -1.0 to 1.0 is considered as normal performance and a Qb-score of ≥ 1.5 is interpreted as divergent. Qb-score between 1.1 to 1.4 is interpreted as slightly divergent. A decrease by ≥ 0.5 of Qb-score is considered as improvement and an increase by ≥ 0.5 as deterioration [25]. The clinical documentation is comprehensive, and results show that QbTest can differentiate between patients and healthy controls and between ADHD and other clinical groups [36, 37]. The psychometric properties with respect to sensitivity (86%) and specificity (83%) have been published [38].

QbActivity includes data from the parameters Time Active, Distance, Area and Microevents.

Time Active is the time (in per cent) the patient has moved more than one centimetre per second (0,4 inches/second). Distance refers to the interval travelled by a reflective marker during the test. Distance is measured in meters. Area is the surface covered by the headband reflector during the test. A Microevent occurs when marker changes its position more than one millimetre since the last Microevent.

QbInattention include the parameters Omission Errors, Reaction Time, Reaction Time Variation and Normalized Variation. An Omission Error occurs when no response is registered to a Target stimulus (the button was not pressed when it should have been). Reaction Time is the average time it takes for the patient to press the response button after the stimuli have been presented. The Reaction Time is measured only when a correct button press is registered. The reported time is measured in milliseconds. Reaction Time Variation is the standard deviation of the Reaction Time. Normalized Variation is the Reaction Time Variation expressed in terms of Reaction Time.

QbImpulsivity includes data from the parameter Commission Error. A Commission Error occurs when a response is registered when the stimulus was a Non-target (the handheld button is pressed when it should not have been pressed). The Error Rate is a measure of the overall accuracy. The Error Rate tells how often the patient has responded incorrectly (pressed the Responder button for non-targets and/or not pressed for targets).

The test time for the QbTest is 20 min and is divided into four five-minute Quartiles, Q1, Q2, Q3 and Q4. The first five-minute Quartile (Q1) is excluded from the analyses, due to many patients have an inconsistent response style in the first five minutes. The analysis is based on the three five-minute Quartiles Q2, Q3 and Q4.

#### Sample size, randomization and masking

A sample of 40 adults was needed to provide 80% power to detect clinically meaningful improvements in performance, using a two-tailed test with alpha = 0.05. An independent research nurse at Centre for Clinical Research, County of Västmanland, generated a simple randomization list. An independent pharmacist labelled and blinded the study medication. The pills were identical in appearance and placed in two bags marked “Day 1” and “Day 2.” The two bags, along with a smaller opaque, sealed envelope containing the identification of the assigned group, were placed in a sealed sequentially numbered identical envelope, one for each participant. The smaller envelope was opened after the participant had completed the study by a medical professional not involved in the study. Neither the research nurse who produced the randomization schedule nor the pharmacist participated in any other aspect of the study. Both investigators and participants were blind to treatment allocation.

#### Procedure

**Fig. 1:**
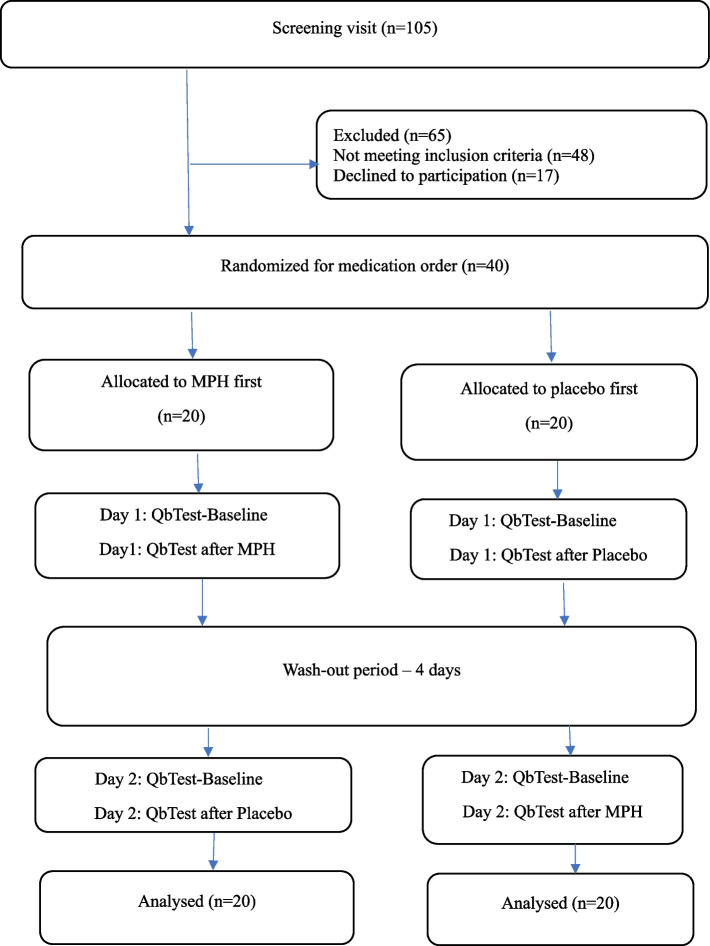
Flowchart of the procedure and randomization of the study. MPH= Methylphenidate, *n*=group size

The participants were consecutively divided on a random basis into two groups, the IR-MPH first group (MPH/placebo group) and the placebo first group (placebo/MPH group). All adults participated on two trial days, with a washout period of 4 days in-between. Upon arrival on the first and second trial day, it was checked for alcohol (ETG 300® Rapid Urine Test Panel; Alcometer Lion 500®—Breath alcohol test) and drug use (Multi-Drug 15 Drugs Rapid Urine Test Panel; ZOP 50® Urine Test Panel; RightSign® Urine). Females were screened for pregnancy (Clearblue®) on the first trial day. Two baselines, one for each trial day, were used to ensure current baseline values and accurately measure the difference between baselines and pills. In one session, the adults received IR-MPH (one single-dose Medikinet® 20 mg Immediate Release pill, first-hand choice of dose during drug trials at the clinic) and in the other session they received placebo (one single-dose placebo). IR-MPH and placebo were counterbalanced across subjects. The study design is shown in Fig. 1. In addition, the participants assessments of expected performance, perceived performance, mental effort and help from the pill were collected. Details and results of the adults’ assessments will be reported elsewhere.

Each session started with a baseline QbTest (without any pill or medication). The adults were shown an instruction video of the QbTest and received additional oral instructions. They were asked to put on the headband and to sit comfortably while holding the response button in their dominant hand and relaxing the other hand on their lap. A practice QbTest trial was given to check if the participant understood the task. Then the adult did the full 20-min QbTest. After the baseline QbTest the adult was given the pill. Eighty minutes elapsed between the adult orally ingesting the pill and began the 2^nd^ QbTest. The QbTest was always scheduled in the morning to prevent potential time-of the day effects that had earlier been reported using the QbTest in children [39].

### Statistical analysis

Medication and placebo efficacy were tested by calculating the differences which we define as the delta scores. Repeated measures ANOVA were used for testing differences in raw test scores between the four conditions (baseline-pre IR-MPH, post IR-MPH, baseline-pre placebo, post placebo) as within-subject factors and calculating changes in performance in the three five-minute Quartiles Q2, Q3 and Q4. Degrees of freedom were corrected according to Greenhouse & Geisser. Bonferroni correction was used for post-hoc comparisons of means. One-way ANOVA was used to analyse differences between groups and gender. Cohen´s d was calculated for effect sizes. Cohen´s d = 0.20 was considered a small effect, d = 0.50 a medium effect and d = 0.80 a large effect. The test–retest reliability between the two baseline conditions (baseline 1^st^ day and baseline 2^nd^ day) was calculated using intra-class correlations (ICC; two-way mixed model, absolute agreement). The statistical software used was IBM SPSS for Windows version 29. All reported p-values were two-tailed, and the level of statistical significance was set at *p* < 0.05.

## Results

Demographic characteristics of the sample are shown in Table 1. Forty adults, 29 females (mean age = 33.5 years) and 11 males (mean age = 34.7 years), were included in the study. None of the participants had earlier been diagnosed or treated for any neuropsychiatric diagnoses. The participants had an average IQ score (mean = 96.8) on the WAIS and no history of substance dependence. Most adults met the criteria for combined presentation of ADHD. There were no statistically significant differences in demographics between the two groups. The adults were given one single-dose 20 mg IR-MPH (i.e., the mean IR-MPH dose was 0.26 [0.17–0.34] mg/kg) and one single-dose placebo on different trial days. There were no differences between groups or gender.

**Table 1:** Clinical and demographic characteristics of the participants when included in the study (*n* = 40)

Variable	MPH/Placebo Group	Placebo/MPHGroup	*p*-value
	(*n* = 20)	(*n* = 20)	
*Gender*			ns
Female (n)	15	14	
Male (n)	5	6	
Age; [mean years (SD)]	33.45 (8.27)	34.20 (11.63)	ns
Height cm; [mean (SD]	168,1 (7.6)	171.6 (9.7)	ns
Weight kg; [mean (SD)]	76.6 (13.7)	82.2 (13.2)	ns
Smoking cigarettes (n)	8	5	ns
Snuff (n)	5	9	ns
GAF^a^; [mean (SD)]	66.55 (6.93)	67.15 (7.04)	ns
ADHD presentation			ns
Hyperactive/impulsive (n)	5	5	
Inattentive (n)	2	2	
Combined (n)	13	13	
ASRS^b^ Hyperactivity; [mean (SD)]	21.70 (7.16)	20.40 (5.54)	ns
ASRS Inattention; [mean (SD)]	24.45 (6.39)	24.25 (5.29)	ns
ASRS Total; [mean (SD)]	46.15 (12.49)	44.15 (10.63)	ns
*QbTest* ^c^ [Z-mean (SD)]			
QbHyperactivity; [mean (SD)]	2.04 (1.27)	1.95 (1.33)	ns
QbInattention; [mean (SD)]	1.77 (0.83)	1.66 (0.81)	ns
QbImpulsivity; [mean (SD)]	0.96 (1.46)	1.35 (1.73)	ns

*SD* Standard Deviation

^a^GAF = Global Assessment of Functioning, assessed by professionals

^b^ASRS = the Adult Self Report Scale; ASRS < 24:Less likely to have ADHD; ASRS < 17:Unlikely to have ADHD

^c^QbTest = The normalized standard scores for the cardinal symptoms hyperactivity, inattention and impulsivity on the QbTest. *p* = probability value. ns = non-significant

### Efficacy measures

Efficacy results are summarized in Table 2. Our primary outcome measures were changes in the cardinal parameters QbActivity, QbInattention and QbImpulsivity on the QbTest. Compared to baseline, the participants´ performance were statistically significant improved in the cardinal parameter QbInattention, F (3, 117) = 38.25, *p* < 0.001, after given IR-MPH (mean diff = 1.14, [95% CI 0.90—1.37], and after placebo (mean diff = 0.60, [95% CI 0.38—0.82)], with the effect sizes (ES) 1.17 and 0.63 respectively. A medication order effect was found. Adults in the placebo/MPH group performed statistically significant better in the cardinal parameter QbInattention on the first trial day compared to the second trial day after given placebo (mean diff = 0.49, [95% CI 0.08—0.91], F (1, 39) = 5.89, *p* < 0.02). IR-MPH improved performance in the cardinal parameters QbActivity (mean diff = 0.81, [95% CI 0.49—1.13], F (2.42, 94.30) = 14.98, *p* < 0.001), and QbImpulsivity (mean diff = 0.46, [95% CI 0.13—0.79], F (3, 117) = 2.79, *p* < 0.04). No statistically significant differences were found between baseline and placebo in the cardinal parameters QbActivity and QbImpulsivity. There were no significant differences between females and males.

**Table 2:** Comparisons of delta scores on the QbTest between baseline, placebo and IR-MPH (*n* = 40)

	Baseline vs Placebo				Baseline vs IR-MPH^a^				IR-MPH^a^ vs Placebo			
Variables	M	SD	p	d^b^	M	SD	p	d^b^	M	SD	p	d^b^
*Cardinal Parameters*												
QbActivity^c^	0.15	0.60	.710	0.12	0.81	0.99	.001	0.60	0.66	1.21	.001	0.83
QbInattention^c^	0.60	0.68	.001	0.63	1.14	0.73	.001	1.17	0.54	0.86	.001	0.77
QbImpulsivity^c^	0.18	1.13	1.000	0.13	0.46	1.04	.047	0.35	0.28	1.52	.259	0.26
*Activity Measures*												
Time Active (%)	3.80	10.67	.182	0.15	10.05	17.46	.005	0.38	6.25	21.29	.071	0.44
Distance (meter)	1.34	4.48	.396	0.09	4.28	6.43	.001	0.37	2.94	8.13	.028	0.54
Area (cm)	7.51	17.36	.066	0.19	19.42	29.12	.001	0.53	11.91	35.27	.039	0.51
*Attention & Impulsive measures*												
Reaction Time (ms)	50.17	6.87	.001	0.44	71.10	55.26	.001	0.72	20.92	78.19	.098	0.67
Reaction Time Variation (ms)	22.82	32.22	.001	0.50	40.12	39.89	.001	0.91	17.30	43.37	.016	0.48
Normalised Variation (%)	2.10	5.78	.162	0.38	3.52	5.23	.001	0.72	1.43	6.86	.197	0.26
Omission Error (%)	5.47	11.71	.032	0.38	16.65	15.53	.001	1.32	11.18	18.18	.001	0.82
Commission Error (%)	0.42	1.24	.229	0.14	0.74	1.73	.058	0.18	0.32	1.98	.249	0.21
Error Rate (%)	4.87	4.35	.001	0.37	1.72	3.24	.011	1.00	3.15	5.14	.001	0.83

^a^Immediate-Release Methylphenidate

^b^d = Effect size (Cohen´s d)

^c^Qb = standard scores

### Clinically significant improvements

An improvement in performance after IR-MPH compared to baseline, and after placebo compared to baseline was considered significant if the Qb-score was decreased ≥ 0.5. A deterioration in performance was considered if the Qb-score was increased ≥ 0.5. The distribution of changes in the cardinal parameters are shown in Table 3. Adults who changed from a slightly divergent score (Qb-score ≥ 1.3) to a normal score (Qb-score ≤ 1.0), of those with a ≥ 0.5 Qb-score reduction, were considered clinically improved. The proportion of adults who were considered clinically improved in the parameters QbInattention, QbActivity and QbImpulsivity after given IR-MPH were 21/36, 20/24 and 12/21, respectively. Corresponding proportions after given placebo were 15/24, 11/12 and 7/14, respectively.

**Table 3:** Distribution of changes in Qb-scores for the cardinal parameters (*n* = 40)

	QbInattention		QbActivity		QbImpulsivity	
	IR-MPH^a^	Placebo	IR-MPH^a^	Placebo	IR-MPH^a^	Placebo
	n (%)	n (%)	n (%)	n (%)	n (%)	n (%)
Improvement^b^	36 (90.0)	24 (60.0)	24 (60.0)	12 (30.0)	21 (52.5)	14 (35.0)
No change	3 (7.5)	12 (30.0)	13 (32.5)	21 (52.5)	10 (25.0)	13 (32.5)
Deterioration^c^	1 (2.5)	4 (10.0)	3 (7.5)	7 (17.5)	9 (22.5)	13 (32.5)

^a^Immediate release methylphenidate

^b^A decrease by ≥ 0.5 Qb-score is considered as improvement

^c^An increase by ≥ 0.5 is considered as deterioration

### Changes in performance during the QbTest

The adults’ performance in the three five-minute Quartiles Q2, Q3 and Q4 during the QbTest are shown in Table 4. Both in baseline and after the adults were given placebo, the activity measures (Time Active, Distance, Area and Microevents) statistically significant increased from Q2 to Q4. After the adults were given IR-MPH, only the activity measure Area statistically significant increased between Q2 to Q4. No statistically significant differences were found between Q2, Q3 and Q4 during baseline, IR-MPH and placebo conditions for the inattention and impulsivity measures.

**Table 4:** Means in the five-minute Quartiles Q2, Q3 and Q4 during the QbTest (*n* = 40)

Parameter	Baseline				IR-MPH^a^				Placebo			
	Q2	Q3	Q4	Sign diff^b^	Q2	Q3	Q4	Sign diff^2^	Q2	Q3	Q4	Sign diff^2^
*Activity measures*												
Time active (%)	28.30	29.30	34.15	Q4 > Q2,Q3	19.82	21.42	21.30	ns	22.27	25.07	27.15	Q4 > Q2;Q3 > Q2
Distance (m)	3.95	4.21	4.85	Q4 > Q2;Q3 > Q2	2.85	3.04	3.19	ns	3.50	3.92	3.90	Q4 > Q2;Q3 > Q2
Area (cm^2^)	18.70	21.02	25.17	Q4 > Q2,Q3;Q3 > Q2	12.22	13.70	14.82	Q4 > Q2	15.70	17.95	18.80	Q4 > Q2;Q3 > Q2
Microevents (mm)	2.31	2.46	2.82	Q4 > Q2,Q3	1.72	1.86	1.93	ns	1.96	2.22	2.27	Q4 > Q2;Q3 > Q2
*Attention & Impulsive measures*												
Reaction Time (ms)	624.75	640.12	631.80	ns	558.07	547.30	555.37	ns	584.07	574.72	574.40	ns
Reaction Time Var (ms)	185.60	191.32	182.67	ns	135.22	132.70	132.85	ns	147.77	146.65	147.70	ns
Normalized Var (%)	29.37	29.60	28.55	ns	27.90	23.97	23.65	ns	24.90	25.25	25.57	ns
Omission Error (%)	21.81	25.34	25.65	ns	7.34	8.56	8.47	ns	17.04	17.12	16.28	ns
Commission Error (%)	2.74	2.17	2.36	ns	1.46	1.59	1.59	ns	1.60	1.37	1.33	ns

^a^Immediate release methylphenidate

^b^Statistically significant differences *p* < 0.05

### Test–retest reliability

Intra-class correlations (ICC) between the two baselines on the first and second trial day for the cardinal parameters QbActivity, QbInattention and QbImpulsivity were 0.92, 0.86 and 0.89, respectively. ICC for the activity parameters Time Active, Distance, Area and Microevents were 0.93, 0.97, 0.95 and 0.96, respectively. ICC for the attention and impulse control measures Reaction Time, Reaction Time Variation, Omission Error and Commission Error were 0.85, 0.79, 0.90 and 0.83, respectively. All correlations were statistically significant at the 0.001 level.

## Discussion

The aim of the present study was to examine the effects of one single-dose IR-MPH compared to one single-dose placebo on performance in ADHD core symptoms during clinical assessment with continuous performance test. In a double-blinded crossover procedure, adults were administered IR-MPH in one session and placebo in the other in randomized order. To our knowledge this is the first study that has examined one single-dose IR-MPH compared to one single-dose placebo in clinical trials with the QbTest in adult patients.

The results of our study show significant improvements in all three core symptoms, and all included parameters except commission error, after intake of one single-dose IR-MPH. Our data are in accordance with results from an earlier study in children [40].

We also noted significant improvements in the cardinal parameter QbInattention and the parameters Reaction Time, Reaction Time Variation and Omission Error after intake of placebo. When we analysed the two groups separately, adults in the placebo/MPH group performed significantly better in the cardinal parameter QbInattention on the first trial day, compared to the second trial day. Adults in the MPH/placebo group also improved their performance in the cardinal parameter QbInattention when given placebo, despite they were verbally informed about the presence of a placebo in one of the trial days. The difference in performance between baseline and placebo may be due to high treatment expectations. According to expectancy theory, placebo effects are mediated by explicit expectancies [41]. These participants had as adults requested a neuropsychiatric assessment for their problems and expected pharmacological treatment. The adults may have expected relief in their symptoms, and this may have made them better to concentrate on the task. Contrary to our hypothesis, placebo showed no effect on neither QbActivity nor QbImpulsivity.

Commission error showed a low effect size in the study probably because this measure has lower sensitivity for adults with ADHD compared to children. In earlier studies, higher rates of Commission Error were found in children compared to adolescents and adults [36, 40, 42]. Pettersson et al., found that only the cardinal parameters QbActivity and QbInattention were significant predictors of clinical diagnosis in adult ADHD [43].

Further, we hypothesized the placebo response would be higher in the beginning and decrease at the end of the test, since adults with ADHD often have difficulties in focusing during a longer period. When we analysed changes in the adults´ performance in the five-minute Quartiles Q2, Q3 and Q4, we noted significantly increases in the activity measures during baseline from Q2 to Q4. Similar results have been reported by Lis et al. [36]. These significantly differences between Q2 and Q4 were also found after the adults were given placebo. As expected, there were no differences found during the IR-MPH condition, due to it was an active substance. Regarding inattention and impulsivity parameters, we noted no differences between Q2 and Q4 in baseline, IR-MPH and placebo conditions during the QbTest. This may indicate that when a person has problems with inattention these are stable over time, compared to problems with activity, which are increasing over time. When we analysed the placebo responders, we found 60% of the adults improved in the cardinal parameter QbInattention (i.e., a decrease by Qb-score ≥ 0.5). However, we must consider that the test situation only lasted for twenty minutes. Further studies with a longer observation period are needed to confirm this.

### Limitations and strengths

One limitation of our study is the unequal distribution of female (72.5%) and male (27.5%) participants. More than half of the patients referred for the neuropsychiatric examination were females. However, there were no differences in gender distribution between adults receiving placebo first or receiving placebo at the second session. In addition, our results showed no differences in performance between females and males during the trial days. Similar proportion in gender (78% females) was reported in a Swedish study [44]. Findings from earlier studies indicate that ADHD is identified more frequently in boys than girls in childhood and more females are identified and become diagnosed in adulthood. However, the differences in prevalence according to gender become far less skewed with age, as well as gender differences in symptoms are limited in adults [8, 45, 46]. Considering the size of the female distribution, hormonal mood changes in females that could have affected the patient’s performance, should have been analysed.

Despite these limitations, strengths of this study are the use of data from a clinical setting. All participants had gone through careful diagnostic procedures, based on validated clinical instruments, including cognitive testing, assigned by trained clinical professionals. Retrospective reports of childhood symptoms were also obtained from the participants´ parents or other relatives. The participants had no history of previous pharmacological treatment regarding their ADHD diagnosis. During the neuropsychiatric assessment period and when the adults were in the research project, they were monitored for alcohol and drug use and females were screened for pregnancy. Before every QbTest, nicotine, snuff and caffeine use were controlled for.

A crossover design was used, where participants were exposed to both test conditions similarly. We used two baselines, one for each day of the trial, to ensure current baseline values and accurately measure the difference between baselines and pills. The two baselines were also used to control for any learning effects, since earlier studies have reported better performance during the second administered CPT [17, 47]. The order of stimulus in the QbTest is randomized in order to prevent practice effects [25]. In addition, we used the baselines to control for carry over effects, although carry over effects are generally less likely in cross-over trials on IR-MPH because of its short pharmacokinetic half-life. Moreover, the test–retest reliability between the two baselines on each trial day was high and in accordance with a previous study [48, 49].

Two studies have found better improvements in participants with most severe baseline symptoms, compared to participants with less severe symptoms [26, 27]. We therefore used a lower cut-off (Qb = 1.3) than the recommended (i.e., Qb-score ≥ 1.5, to indicate a divergent score [25]). The lower cut-off was chosen to avoid regression to the mean effects.

## Conclusions

The results of this study demonstrated improvements in performance in the core symptom inattention, after given one single-dose placebo. This implies that knowledge of the effect of placebo can be useful when treating patients with predominantly inattentive presentation. In addition, we noted that activity problems increased during baseline and after placebo intake during the QbTest while inattention and impulsivity difficulties remained at the same level during the QbTest. This finding could be relevant in the interpretation of results from the QbTest with new patients.

## Data Availability

Research data that support the findings of this study are not public available due to protection of participants´ confidentiality. Anonymized dataset can be created and made available upon reasonable requests from the corresponding author.

## Abbreviations

ADHD: Attention-deficit/hyperactivity disorder
CPT: Continuous performance test
IR-MPH: Immediate released Methylphenidate
QbTest: The Quantified behavioral Test
